# CRISPR-Cas9 base editing of pathogenic CaMKII**δ** improves cardiac function in a humanized mouse model

**DOI:** 10.1172/JCI175164

**Published:** 2024-01-02

**Authors:** Simon Lebek, Xurde M. Caravia, Leon G. Straub, Damir Alzhanov, Wei Tan, Hui Li, John R. McAnally, Kenian Chen, Lin Xu, Philipp E. Scherer, Ning Liu, Rhonda Bassel-Duby, Eric N. Olson

**Affiliations:** 1 Department of Molecular Biology and; 2 Hamon Center for Regenerative Science and Medicine, University of Texas Southwestern Medical Center, Dallas, Texas, USA.; 3 Department of Internal Medicine II, University Hospital Regensburg, Regensburg, Germany.; 4 Touchstone Diabetes Center and; 5 Quantitative Biomedical Research Center, Peter O’Donnell Jr. School of Public Health, University of Texas Southwestern Medical Center, Dallas, Texas, USA.

**Keywords:** Cardiology, Cardiovascular disease, Gene therapy, Mouse models

## Abstract

Cardiovascular diseases are the most common cause of worldwide morbidity and mortality, highlighting the necessity for advanced therapeutic strategies. Ca^2+^/calmodulin-dependent protein kinase IIδ (CaMKIIδ) is a prominent inducer of various cardiac disorders, which is mediated by 2 oxidation-sensitive methionine residues within the regulatory domain. We have previously shown that ablation of CaMKIIδ oxidation by CRISPR-Cas9 base editing enables the heart to recover function from otherwise severe damage following ischemia/reperfusion (IR) injury. Here, we extended this therapeutic concept toward potential clinical translation. We generated a humanized *CAMK2D* knockin mouse model in which the genomic sequence encoding the entire regulatory domain was replaced with the human sequence. This enabled comparison and optimization of two different editing strategies for the human genome in mice. To edit *CAMK2D* in vivo, we packaged the optimized editing components into an engineered myotropic adeno-associated virus (MyoAAV 2A), which enabled efficient delivery at a very low AAV dose into the humanized mice at the time of IR injury. *CAMK2D*-edited mice recovered cardiac function, showed improved exercise performance, and were protected from myocardial fibrosis, which was otherwise observed in injured control mice after IR. Our findings identify a potentially effective strategy for cardioprotection in response to oxidative damage.

## Graphical abstract

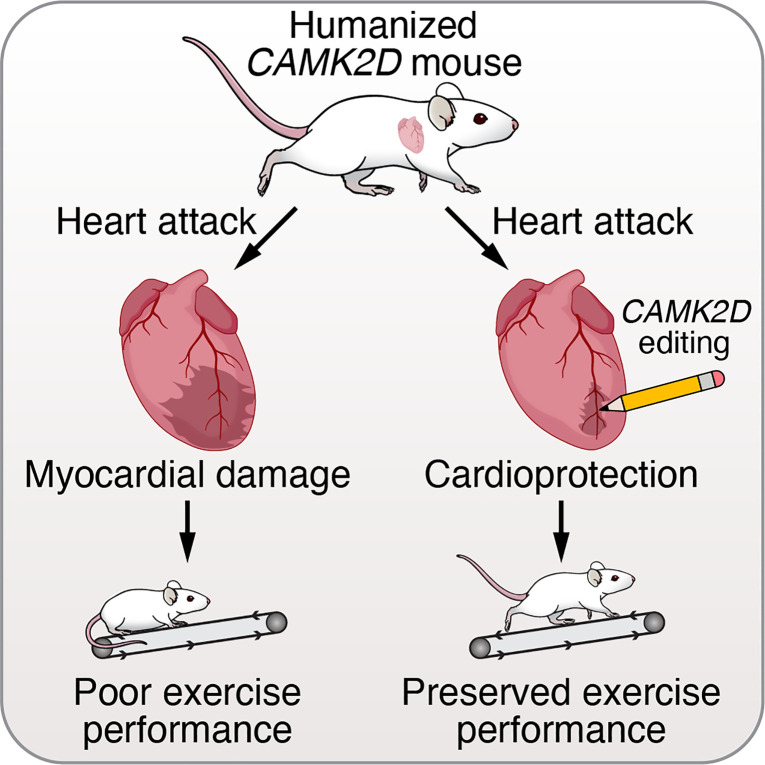

## Introduction

Cardiovascular diseases, in particular coronary artery heart disease with subsequent myocardial infarction, are the most common cause of death worldwide, highlighting the necessity for advanced therapeutic strategies (1). Chronic overactivation of Ca^2+^ /calmodulin-dependent protein kinase IIδ (CaMKIIδ) has been shown to be a central indicator and inducer of cardiac disease (2–10). While regulating cellular homeostasis and signaling at normal activation levels, sustained increased CaMKIIδ activation has been linked to impaired excitation-contraction coupling, disturbances in cellular Ca^2+^ handling, inflammation, apoptosis, and fibrosis, all of which impair cardiac function (2, 4, 5, 8–14). Accordingly, CaMKIIδ overactivation has been linked to myocardial infarction and ischemia/reperfusion (IR) injury, heart failure, arrhythmias, cardiac hypertrophy, and sleep-disordered breathing (2, 3, 6–11, 15, 16). Mechanistically, oxidation of 2 methionine residues at position 281 and 282 has been shown to activate CaMKIIδ by preventing association of the autoinhibitory region with the catalytic domain (3, 10, 15–17). Indeed, *Camk2d* knockin mice, in which both oxidation-sensitive methionines were replaced with oxidation-resistant valines in the germline, were protected from severe cardiac damage (3, 15, 16). These studies suggested that preventing CaMKIIδ oxidation may offer a cardioprotective benefit to heart disease.

As knockin technology is not suitable for potential clinical application, we have previously developed a CRISPR-Cas9 adenine base editing strategy to ablate CaMKIIδ oxidation in vivo in adult mice (10). Base editing involves a fusion protein of Cas9 nickase or inactive Cas9 fused to a deaminase domain, which is directed to a genome sequence by a single-guide RNA (sgRNA) (18–23). Adenine base editing (ABE) allows the precise conversion of adenine to guanine nucleotides without introducing double-stranded DNA breaks (10, 18–25). As methionines are encoded by ATG codons, we utilized ABE to convert the 2 methionine residues at position 281 and 282 to valines encoded by GTG codons in adult mice, thereby rendering CaMKIIδ insensitive to oxidative activation (10). Administration of *Camk2d* editing components at the day of IR injury to adult mice achieved successful editing and enabled the heart to recover function from otherwise severe damage (10). *CAMK2D* gene editing may thus represent a permanent and advanced strategy for heart disease therapy (10).

While our previous work demonstrated a potential intervention to protect the heart from IR damage, the approach has several limitations that could diminish its therapeutic application. First, the previous study used the mouse sgRNA sequence targeting the mouse genomic sequence of the CaMKIIδ oxidation sites, which is different from the human sequence. Therefore, the efficacy of the human sgRNA in vivo remains to be determined. Second, we administered a relatively high adeno-associated virus (AAV) dose to adult mice (10). However, high AAV doses have been associated with serious adverse effects, including acute liver damage, thrombocytopenia, and immunological responses with preexisting antibodies (18, 26, 27). Large-scale AAV production also poses substantial challenges. It is thus imperative to deliver AAV at the lowest effective dose. In addition, we performed a broad-windowed ablation of the oxidative activation sites of CaMKIIδ that contained bystander-editing in vivo (10).

The aim of the present study was to further advance our previous strategy of rendering CaMKIIδ resistant to oxidative activation toward a potential therapy for human cardiac disease. Therefore, we generated a *CAMK2D* knockin mouse model to humanize the regulatory domain of CaMKIIδ, which allowed the use of human sgRNA sequences in vivo in mice. We made efforts to reduce off-target editing and to decrease the AAV dose for delivery of the gene editing components in vivo. We further compared different editing patterns in vivo following IR, as narrow-windowed and bystander-free editing of only one methionine-encoding adenine may potentially be sufficient to confer cardioprotection (10, 17). In addition, we tested whether *CAMK2D* editing improved exercise performance following IR.

## Results

### Development of an optimized gene editing strategy in human induced pluripotent stem cells to reduce off-target editing.

**Figure 1:**
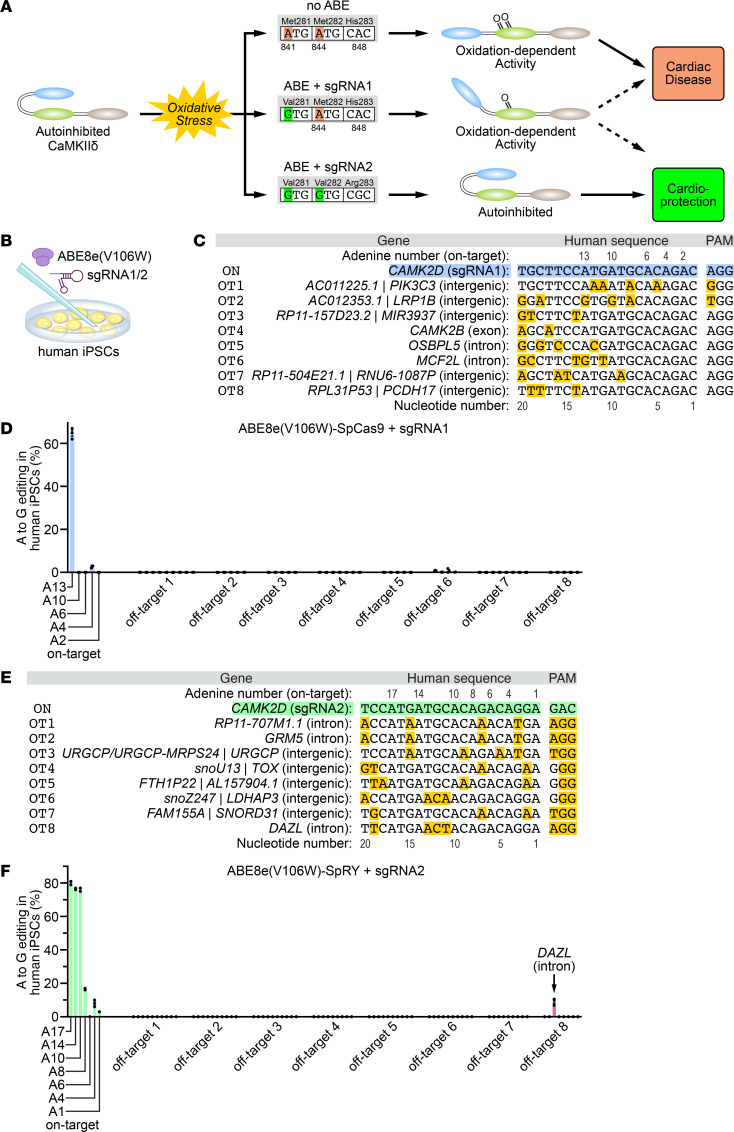
Analysis of on- and off-target editing in human iPSCs. (**A**) Schematic showing the structure of CaMKIIδ with its 3 domains (blue, catalytic domain; green, regulatory domain; brown, association domain). Upon oxidative stress, 2 critical methionine residues at position 281 and 282 become oxidized, thereby preventing association of the regulatory with the catalytic domain, and resulting in cardiac disease. We deployed 2 ABE strategies to ablate either 1 (sgRNA1) or both (sgRNA2) oxidative activation sites of CaMKIIδ. (**B**) Human iPSCs were nucleofected with ABE8e(V106W) and either sgRNA1 or sgRNA2. (**C**) Sequence of sgRNA1 (*CAMK2D* on-target, ON) and the corresponding DNA and PAM sequences of the top 8 predicted potential off-target sites (OT), as predicted by CRISPOR. Nucleotides highlighted in yellow are different from sgRNA1. (**D**) Percentage of adenine (A) to guanine (G) editing for all adenines within the on- and off-target sites (ordered from 5′ to 3′) following adenine base editing with ABE8e(V106W)-SpCas9 + sgRNA1 (*n* = 3). (**E**) Sequence of sgRNA2 (*CAMK2D* on-target, ON) and the corresponding DNA and PAM sequences of the top 8 predicted potential off-target sites, as predicted by CRISPOR. Nucleotides highlighted in yellow are different from sgRNA2. (**F**) Percentage of adenine (A) to guanine (G) editing for all adenines within the on- and off-target sites (ordered from 5′ to 3′) following adenine base editing with ABE8e(V106W)-SpRY + sgRNA2 (*n* = 3). All data are individual data points with mean ± SEM. Replicates are human iPSCs following 3 independent nucleofections.

In our prior studies, we performed adenine base editing of methionine-encoding ATG codons of *CAMK2D* in human induced pluripotent stem cells (iPSCs) with high editing efficiencies. However, we observed off-target editing of 29.5% at an intronic site in the *DAZL* gene (10). Thus, to further develop an optimized base editing strategy for *CAMK2D* with reduced off-target editing, we used an engineered ABE8e variant (TadA-8e V106W) that causes less off-target editing (Figure 1, A and B) (10, 19). Here, we compared 2 different editing strategies targeting the oxidative activation sites of CaMKIIδ (Figure 1A). First, we deployed sgRNA1 that places *CAMK2D* c.A841 (p.M281) in protospacer position 13 (counted from the 5′ end of the protospacer adjacent motif [PAM] sequence) together with ABE8e(V106W) fused to SpCas9 nickase (targeting NGG PAMs; Figure 1C) (20). Following nucleofection of human iPSCs, we observed a mean editing efficiency of 64.7% ± 1.5% for c.A841G (p.M281V) (Figure 1, C and D). Notably, we detected no marked editing of c.A844 (p.M282) (Figure 1D) or any off-target editing at the top 8 predicted potential off-target sites in the human genome (Figure 1D).

We further analyzed sgRNA2 combined with ABE8e(V106W) fused to the engineered variant SpRY nickase that targets NRN (and NYN PAMs to a lesser extent; Figure 1E) (21). When using sgRNA2, adenines within the methionine-281 (c.A841) and -282 (c.A844) codons are placed in protospacer positions 17 and 14, respectively. Nucleofection of human iPSCs and subsequent DNA sequencing revealed an editing efficiency of 80.3% ± 0.7% for c.A841G (p.M281V), 76.7% ± 0.3% for c.A844G (p.M282V), and 76.3% ± 0.7% for c.A848G (p.H283R) (Figure 1F). Deep amplicon sequencing of the top 8 predicted potential off-target sites revealed adenine to guanine editing of 9.2% ± 1.1% in an intronic region of the *DAZL* gene, which was greatly reduced compared with the 29.5% off-target editing with the previous editing strategy (10). No other off-target editing was detected (Figure 1F).

### Editing oxidation-sensitive methionines of CaMKIIδ in a humanized mouse after IR.

**Figure 2:**
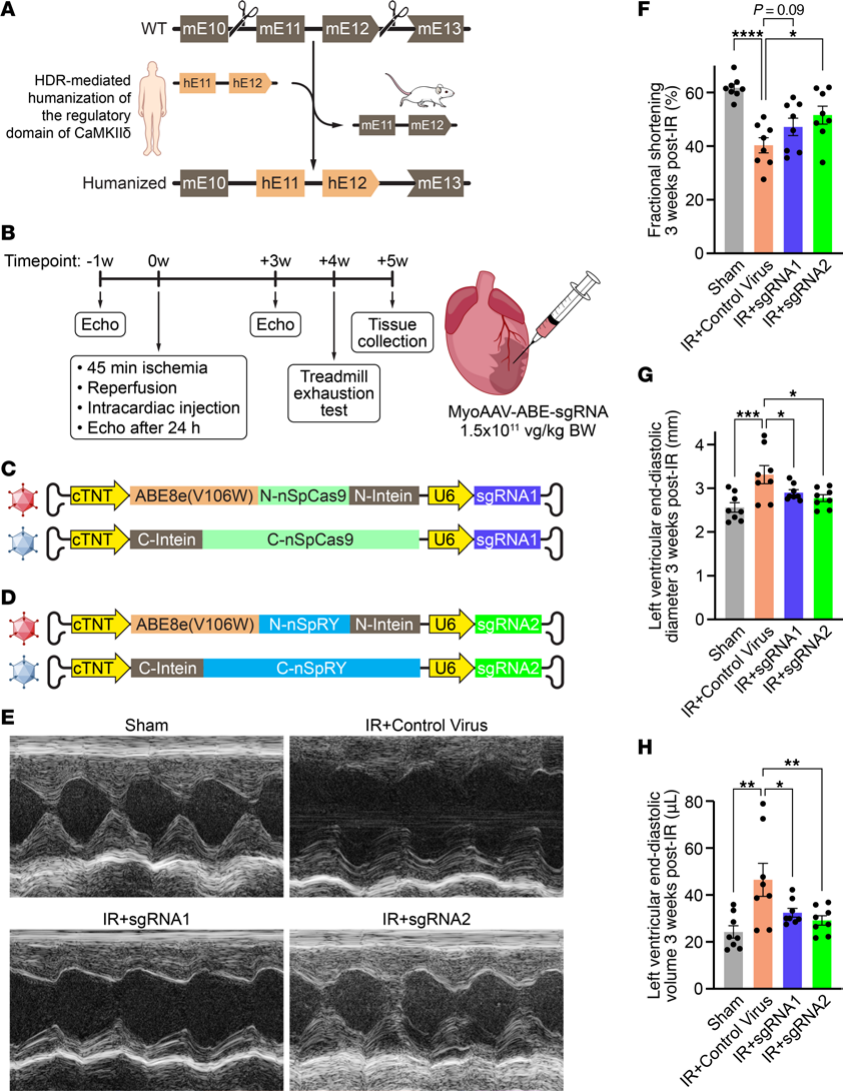
Editing *CAMK2D* in a humanized mouse model upon IR injury. (**A**) Schematic showing humanization of the mouse sequence of the regulatory domain of CaMKIIδ, which is encoded by exons 11 and 12. (**B**) Flowchart showing the experimental design for subjecting 12-week-old humanized *CAMK2D* female and male mice to IR. Cardiac function was assessed 1 week before as well as 24 hours and 3 weeks after IR by echocardiography. At 4 weeks after IR, mice were subjected to a treadmill exhaustion test. At 5 weeks after IR, mice were sacrificed, and tissue was collected for further analyses. (**C**) Illustration of the split-MyoAAV 2A that was used to deliver ABE8e(V106W)-SpCas9 + sgRNA1 in vivo. (**D**) Illustration of the split-MyoAAV 2A that was used to deliver ABE8e(V106W)-SpRY + sgRNA2 in vivo. (**E**) Representative M-mode traces of hearts from mice subjected to either sham, IR+control virus, IR+sgRNA1 or IR+sgRNA2 (echocardiography; 3 weeks after IR; in total *n* = 8 per group). (**F**) Mean fractional shortening 3 weeks after IR (*n* = 8 per group). (**G**) Mean LVIDd 3 weeks after IR (*n* = 8 per group). (**H**) Mean LVIVd 3 weeks after IR (*n* = 8 per group). All data are individual data points with mean ± SEM and all replicates are individual mice. Statistical comparisons are based on 1-way ANOVA posthoc corrected by Holm-Šidák (**F**–**H**); *P < 0.05, **P < 0.01, ***P < 0.001, ****P < 0.0001.

Efficient CRISPR-Cas9 gene editing is critically dependent on the sgRNA that corresponds to a specific target sequence in the genome (18, 20). While the amino acid sequence of CaMKIIδ is highly conserved across species, the DNA sequence differs. For example, for sgRNA1, there is a 10% mismatch between the human and corresponding mouse sequence, which prevents highly efficient gene editing between species (18–20, 22–25). Thus, to enable possible translation to the human gene, we humanized the entire regulatory domain of the mouse CaMKIIδ by exchanging exons 11 and 12, intron 11, and portions of the flanking introns 10 and 12 with the human sequence, using CRISPR-Cas9-mediated homology-directed repair (HDR) (Figure 2A and Supplemental Figure 1; supplemental material available online with this article; https://doi.org/10.1172/JCI175164DS1). This humanized mouse model enables deployment of gene editing strategies optimized for the human genome in vivo in mice. Compared with WT littermates, homozygous humanized mice showed normal body and heart weight at 12 weeks of age (Supplemental Figure 2, A and B). Sequencing of cDNA showed that the humanized exons 11 and 12 were properly spliced between the mouse exons 10 and 13 (Supplemental Figure 2C). Western blot analyses revealed comparable CaMKII protein expression between mice with WT or humanized *CAMK2D* (Supplemental Figure 2, D and E). Accordingly, both genotypes had similar CaMKII activity at baseline, making the humanized *CAMK2D* mouse a suitable model for in vivo gene editing (Supplemental Figure 2F).

In the present study, we used female and male mice that were homozygous for the humanized *CAMK2D* knockin and both sexes were represented equally within each group. A week before IR surgery, mice were subjected to baseline echocardiography (Figure 2B and Supplemental Figure 3). Importantly, all mice showed normal fractional shortening, left ventricular end–diastolic internal diameter (LVIDd), and left ventricular end–diastolic internal volume (LVIVd) with no differences across all groups (Supplemental Figure 3). At 12 weeks of age, mice were subjected to either 45 minute open chest surgery (sham) or to 45 minute ligation of the left anterior descending coronary artery (IR; Figure 2B). To deliver the *CAMK2D* editing components in vivo into the heart, we used AAV with the myotropic AAV2/MyoAAV 2A serotype (28). Due to the limited packaging size of AAVs, which is exceeded by the ABE expression cassette, we used split-intein trans-splicing systems (Figure 2, C and D). A pair of MyoAAVs carried ABE8e(V106W)-SpCas9 + sgRNA1 (Figure 2C). The second pair of MyoAAVs carried ABE8e(V106W)-SpRY + sgRNA2 (Figure 2D). Cardiomyocyte-specificity was ensured by using the cardiac troponin T (cTnT) promoter to drive the expression of the editing components. Mice subjected to IR received a single intracardiac injection after reperfusion of either a nonediting control MyoAAV (IR+control virus), MyoAAV-ABE-sgRNA1 (IR+sgRNA1), or MyoAAV-ABE-sgRNA2 (IR+sgRNA2). Each mouse received a total virus dosage of 1.5 × 10^11^ vg/kg body weight (equal amounts of N- and C-term for IR+sgRNA1 and IR+sgRNA2).

After 24 hours, all mice subjected to IR showed a substantial decline in cardiac function (Supplemental Figure 4). Compared with sham mice with 57.7% ± 1.1% fractional shortening, we observed a decrease in fractional shortening to 32.2% ± 2.3% in IR+control virus (*P* < 0.0001), 35.6% ± 2.4% in IR+sgRNA1 (*P* < 0.0001), and 37.0% ± 0.9% in IR+sgRNA2 (*P* < 0.0001) (Supplemental Figure 4B). Moreover, LVIDd increased from 2.3 ± 0.1 mm in sham mice, to 3.3 ± 0.1 mm in IR+control virus (*P* < 0.0001), 3.1 ± 0.1 mm in IR+sgRNA1 (*P* = 0.0002), and 2.9 ± 0.1 mm in IR+sgRNA2 (*P* = 0.004) mice (Supplemental Figure 4C). Similarly, LVIVd was also significantly increased in all groups subjected to IR compared with sham mice (Supplemental Figure 4D).

Three weeks after IR, *CAMK2D*-edited mice displayed a significantly improved fractional shortening from 40.3% ± 2.8% in IR+control virus to 51.6% ± 3.4% in IR+sgRNA2 (*P* = 0.02) (Figure 2, E and F). Notably, mice subjected to IR+sgRNA1 only showed a nonsignificant intermediate recovery to 47.2% ± 3.2% (*P* = 0.09). LVIDd decreased from 3.3 ± 0.2 mm in IR+control virus to 2.9 ± 0.1 mm (*P* = 0.03) and 2.8 ± 0.1 mm (*P* = 0.01) in IR+sgRNA1 and IR+sgRNA2, respectively (Figure 2G). Similarly, LVIVd was almost normalized from 46.5 ± 7.0 μL in IR+control virus to 32.4 ± 1.9 μL and 29.2 ± 2.0 μL in IR+sgRNA1 (*P* = 0.02) and IR+sgRNA2 (*P* = 0.0095), respectively (Figure 2H).

### Functional and mechanistic analyses in CAMK2D-edited mice after IR.

**Figure 3:**
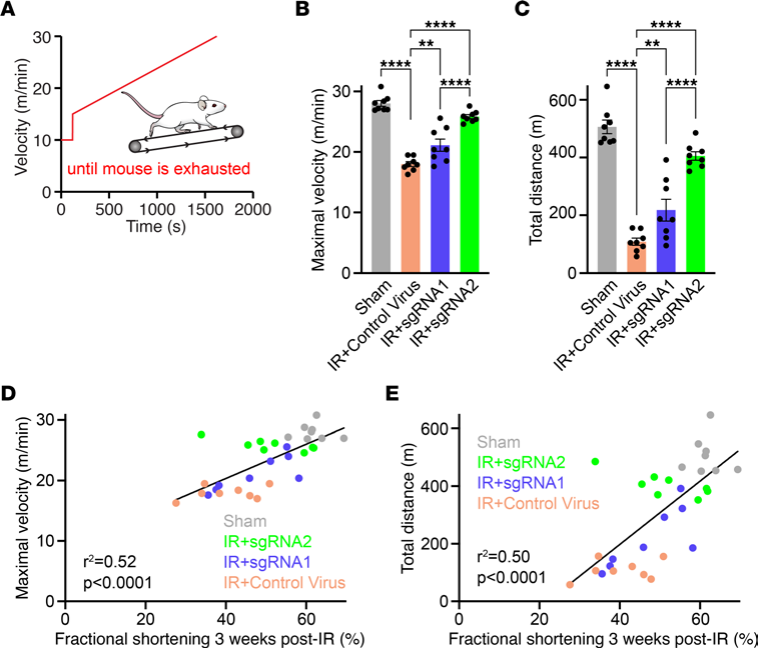
*CAMK2D*-edited mice show improved exercise performance after IR. (**A**) Protocol used for the treadmill exhaustion test. (**B**) Mean maximal velocity achieved on the treadmill prior to exhaustion (*n* = 8 per group). (**C**) Mean total distance achieved on the treadmill prior to exhaustion (*n* = 8 per group). (**D**) Linear regression analysis of fractional shortening and the corresponding maximal velocity achieved on the treadmill (*n* = 8 per group, *n* = 32 in total). (**E**) Linear regression analysis of fractional shortening and the corresponding total distance achieved on the treadmill (*n* = 8 per group, *n* = 32 in total). All data are individual data points with mean ± SEM and all replicates are individual mice. Statistical comparisons are based on 1-way ANOVA posthoc corrected by Holm-Šidák (**B** and **C**) and linear regression analysis (**D** and **E**); **P < 0.01, ****P < 0.0001.

As exercise performance depends on both skeletal muscle and cardiovascular function, we reasoned that recovery of cardiac function should improve exercise performance after IR. Thus, all mice were subjected to a treadmill exhaustion test 4 weeks after IR, according to a standardized protocol (10). As expected, mice treated with a nonediting control virus achieved a substantially lower maximal velocity of 18.0 ± 0.4 m/min compared with 28.0 ± 0.5 m/min in sham-treated mice (*P* < 0.0001) (Figure 3, A and B). Compared with IR+control virus, *CAMK2D*-edited mice showed a higher maximal velocity of 21.1 ± 1.0 m/min (*P* = 0.001) and 25.9 ± 0.3 m/min (*P* < 0.0001) with IR+sgRNA1 and IR+sgRNA2, respectively (Figure 3B). Notably, mice treated with sgRNA2 showed a significantly higher maximal velocity than mice treated with sgRNA1 (*P* < 0.0001). Accordingly, the total distance run on the treadmill was approximately 4.6-fold shorter in mice treated with a control virus (109.2 ± 12.3 m) compared with sham-treated mice (506.4±23.6 m, *P* < 0.0001) (Figure 3C). Compared with IR+control virus, *CAMK2D*-edited mice achieved a longer distance of 218.1 ± 37.3 m in IR+sgRNA1 (*P* = 0.003) and an even higher recovery with 405.4 ± 14.7 m in mice subjected IR+sgRNA2 (*P* < 0.0001 versus IR+control virus, *P* < 0.0001 versus IR+sgRNA1) (Figure 3C). Moreover, we found a significant correlation between fractional shortening and both the maximal velocity (*P* < 0.0001, r^2^ = 0.52) and the total distance (*P* < 0.0001, r^2^ = 0.50) achieved on the treadmill, further validating our findings (Figure 3, D and E).

**Figure 4:**
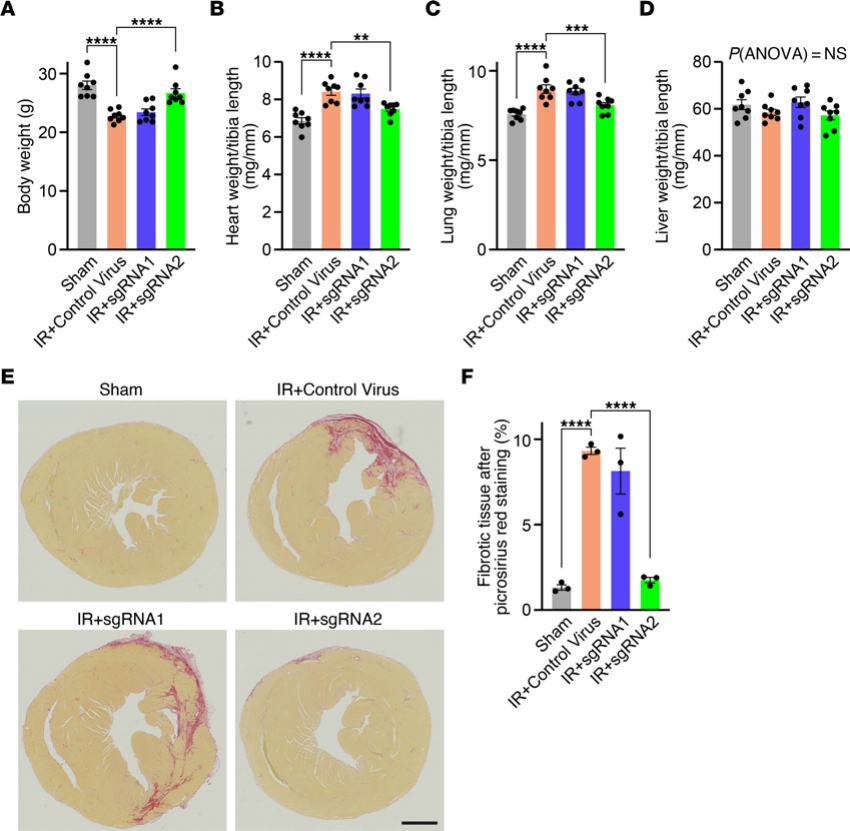
Normal organ weights and prevention of fibrosis in sgRNA2-treated mice after IR. (**A**) Mean body weight for mice subjected to sham, IR+control virus, IR+sgRNA1 or IR+sgRNA2 (*n* = 8 per group). (**B**) Mean heart weight normalized to tibia length (*n* = 8 per group). (**C**) Mean lung weight normalized to tibia length (*n* = 8 per group). (**D**) Mean liver weight normalized to tibia length (*n* = 8 per group). (**E**) Representative picrosirius red staining of transverse cardiac sections from all groups (scale bar: 1,000 μm). (**F**) Mean percentage of fibrotic tissue (*n* = 3 per group). All data are individual data points with mean ± SEM and all replicates are individual mice. Statistical comparisons are based on 1-way ANOVA post-hoc corrected by Holm-Šidák (**A**–**D** and **F**); **P < 0.01, ***P < 0.001, ****P < 0.0001.

Five weeks after IR, all mice were sacrificed for further analyses. Mice treated with the control virus after IR showed a reduced body weight of 22.8 ± 0.4 g compared with sham mice, with 28.0 ± 0.7 g (*P* < 0.0001), which is frequently observed in patients with critical illness (Figure 4A). Interestingly, only mice treated with sgRNA2 after IR recovered in body weight to 26.7 ± 0.7 g (*P* < 0.0001) (Figure 4A). Additionally, control mice after IR showed increased heart and lung weights compared with sham, which were both significantly improved in mice treated with sgRNA2 but not with sgRNA1 after IR (Figure 4, B and C). Importantly, there was no difference in liver weight across all groups, indicating that there was no severe liver failure due to the potential liver toxicity of AAV (Figure 4D). Analysis of picrosirius red–stained cardiac sections revealed an approximately 7.0-fold increase in fibrotic area in control mice after IR, which was 9.3% ± 0.2% in IR+control virus compared with 1.3% ± 0.2% in sham (*P* < 0.0001) (Figure 4, E and F). Importantly, only treatment with sgRNA2 but not with sgRNA1 resulted in a substantially reduced region of fibrotic tissue of 1.8% ± 0.2% in IR+sgRNA2 (*P* < 0.0001) and only a small residual fibrotic area was detectable (Figure 4, E and F).

### Analysis of editing efficiency in humanized mice upon IR.

**Figure 5:**
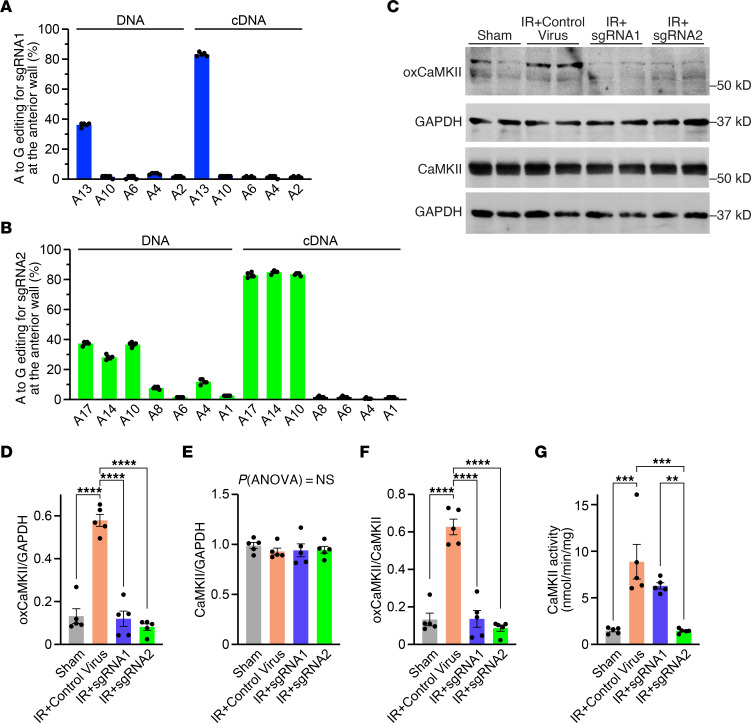
Analysis of editing efficiency. (**A**) Percentage of adenine (A) to guanine (G) editing at the DNA and cDNA level in the anterior cardiac wall of mice following treatment with ABE8e(V106W)-SpCas9 + sgRNA1 (*n* = 5 per group). (**B**) Percentage of adenine (A) to guanine (G) editing at the DNA and cDNA level in the anterior cardiac wall of mice following treatment with ABE8e(V106W)-SpRY + sgRNA2 (*n* = 5 per group). (**C**) Western blot analysis of oxidized CaMKII, total CaMKII, and GAPDH in mice subjected to either sham, IR+control virus, IR+sgRNA1 or IR+sgRNA2 (in total *n* = 5 per group). (**D**) Mean densitometric analysis for oxidized CaMKII normalized to GAPDH (*n* = 5 per group). (**E**) Mean densitometric analysis for total CaMKII normalized to GAPDH (*n* = 5 per group). (**F**) Mean densitometric analysis for oxidized CaMKII normalized to total CaMKII (*n* = 5 per group). (**G**) Mean CaMKII activity for all groups (*n* = 5 per group). All data are individual data points with mean ± SEM and all replicates are individual mice. Statistical comparisons are based on 1-way ANOVA post-hoc corrected by Holm-Šidák (**D**–**G**); **P < 0.01, ***P < 0.001, ****P < 0.0001.

The editing efficiency was assessed in myocardial samples of the anterior cardiac wall, as this is the area of interest that was injured by IR and where the editing components were injected. In fact, we previously found that editing was restricted to the region of the intracardiac injection (10). In mice treated with sgRNA1, sequencing analyses revealed an editing efficiency of 36.2% ± 0.6% at the DNA and 83.2% ± 0.6% at the cDNA level for c.A841G (p.M281V), while no marked editing of c.A844 (p.M282) was detected (Figure 5A). In mice treated with sgRNA2, we measured an editing efficiency of 37.0% ± 0.5%, 27.8% ± 0.7%, and 36.4% ± 0.7% at the DNA level, and 82.8% ± 0.7%, 84.8% ± 0.5%, and 83.4% ± 0.4% at the cDNA level for c.A841G (p.M281V), c.A844G (p.M282V), and c.A848G (p.H283R), respectively (Figure 5B). The editing efficiency differs between the DNA and cDNA level since only DNA of cardiomyocytes is targeted when using a cTnT promoter and cardiomyocytes represent only approximately 40% of all cells in the heart (29). However, most of the cardiac CaMKIIδ is expressed by cardiomyocytes, explaining the high editing efficiency at the cDNA level.

As expected, Western blot analyses revealed an approximately 4.7-fold increase in oxidized CaMKII in control mice post-IR, but not when the oxidative activation sites were ablated using ABE (Figure 5, C–F). There was no difference in total CaMKII expression across all groups (Figure 5, C and E). When normalized to total CaMKII expression, CaMKII oxidation levels increased from 0.13 ± 0.03 in sham to 0.63 ± 0.04 in IR+control virus (*P* < 0.0001; based on densitometric analysis) (Figure 5F). In contrast, we observed only minimal levels of oxidized CaMKII with 0.14 ± 0.04 and 0.09 ± 0.02 in IR+sgRNA1 and IR+sgRNA2, respectively (*P* < 0.0001 for both groups versus IR+control virus). This residual signal can either represent unedited CaMKIIδ, oxidized CaMKIIγ, or unspecific background. Ablation of just methionine-281 with sgRNA1 was sufficient to decrease the binding affinity of the antibody recognizing oxidized CaMKII, which is in accordance with our previous data in iPSC-cardiomyocytes and also with other studies analyzing posttranslational modification of an individual amino acid (10). Measurement of CaMKII activity showed an approximately 6.0-fold increased signal of 8.9 ± 1.9 nmol/min/mg in IR+control virus compared with 1.5 ± 0.1 nmol/min/mg in sham (*P* = 0.0001) (Figure 5G). Importantly, only treatment with sgRNA2, which ablates both oxidative activation sites, could normalize CaMKII activity to 1.5 ± 0.1 nmol/min/mg after IR (*P* = 0.0001 versus IR+control virus), which was substantially lower than 6.3 ± 0.3 nmol/min/mg in IR+sgRNA1 (*P* = 0.002) (Figure 5G).

## Discussion

The aim of the present study was to build on our previous work of rendering CaMKIIδ resistant to oxidative activation and to move one step closer to potential translation as a therapeutic strategy for IR injury (10). Highly efficient CRISPR-Cas9 gene editing is critically dependent on the sgRNAs that are designed to bind a specific DNA sequence (10, 18, 24). Even though the amino acid sequence is highly conserved between human and mouse, the *CAMK2D* DNA sequence varies between species, precluding the use of the human sgRNAs in vivo in WT mice. For this reason, we generated a humanized *CAMK2D* knockin mouse model, where the entire regulatory domain (exons 11 and 12 with parts of the flanking introns) was replaced with the human sequence. This humanized mouse model allowed the deployment of the gene editing strategies optimized for humans in vivo in mice.

Up to now, it was unclear whether ablation of both oxidative activation sites of CaMKIIδ (methionine-281 and methionine-282) was required to confer resistance to IR injury or whether the full cardioprotective effect could be achieved by modifying a single residue. Therefore, in the current work, we applied and compared 2 different *CAMK2D* editing strategies in vivo that we previously identified as lead-candidates in human iPSCs in vitro (10). One editing strategy represents a narrow-windowed approach that only modifies methionine-281 (M281V; sgRNA1). The other strategy introduces a broader editing pattern that modifies both methionines together with a bystander mutation (MMH281/282/283VVR; sgRNA2). We found that ablating both oxidative activation sites (sgRNA2) conferred a higher degree of cardioprotection upon IR than ablation of just methionine-281 (sgRNA1). This difference is less pronounced in the echocardiographic analyses, which may be due to technical variability. We conclude that the presence of 1 oxidized methionine is still sufficient to disrupt the reassociation of the autoinhibitory region with the catalytic domain of CaMKIIδ, even though to a lesser extent than the presence of both oxidized methionines. This conclusion is further supported by our data showing a level of CaMKII activity in sgRNA1-treated mice intermediate between mice treated with a control virus or with sgRNA2. Future studies will include work comparing specific editing of the CaMKIIδ oxidative activation sites with cardiomyocyte-restricted ablation of the complete enzyme, which could also be achieved with CRISPR-Cas9 technology. As CaMKIIδ is an important regulator of cardiac physiology at a normal activation level, complete ablation of this enzyme might not be the most beneficial strategy, but this remains to be tested (14, 30).

Base editing may also cause off-target editing, and, indeed, we previously observed off-target editing at an intronic site in the *DAZL* gene when using ABE8e-SpRY + sgRNA2. Therefore, we further developed and optimized our editing strategy by introducing a V106W substitution into the TadA domain of the base editor, which has been shown to reduce off-target editing (19). Indeed, we observed no marked off-target editing at any of the top 8 potential off-target sites when using ABE8e(V106W) + sgRNA1. We were further able to substantially decrease off-target editing in the *DAZL* gene — previously 29.5% to 9.2% — now when using ABE8e(V106W)-SpRY + sgRNA2 (10). This off-target site is located in an intronic region of *DAZL*, meaning that this edit would not be expressed in an mRNA transcript. Plus, using a cTnT promoter restricts potential off-target editing to cardiomyocytes, where DAZL expression is very low (31). We thus think that the clinical consequences of editing *DAZL* would be minimal. Before a potential first-in-human clinical trial, a broader analysis of potential off-target editing will be necessary (e.g., whole-genome sequencing of human myocardial biopsies treated with *CAMK2D* editing). We did not assess potential off-target editing in the mouse genome. As the DNA sequences differ between the human and mouse genomes, also the potential genomic off-target sites differ across species and are difficult to compare. For example, the off-target sequence in the human *DAZL* gene does not exist in the mouse *Dazl* gene. Interpreting the relevance of potential off-target editing in the mouse genome following treatment with a human sgRNA would be even more difficult.

Another challenge of efficient in vivo gene editing is the choice of the delivery modality. To date, most approaches have relied on AAV, which shows good infectivity of cardiac tissue (18, 24, 25, 29, 32). However, systemic administration of high AAV doses has been associated with serious adverse effects, including acute liver damage, thrombocytopenia, and immunological responses with preexisting antibodies (18, 26, 27). Large-scale AAV production also poses substantial challenges. It is thus imperative to keep the viral dose as low as possible or to deploy other delivery strategies like lipid nanoparticles or virus-like particles (18, 28, 33–35). We opted for an engineered AAV vector with a modified capsid protein following 2 generations of directed evolution (MyoAAV 2A) (28). Compared with AAV9, the transduction efficiency of MyoAAV 2A has been shown to be approximately 17-fold higher in the heart and approximately 2.5 times lower in the liver following systemic administration in mice (28). To achieve therapeutic thresholds in muscle tissue with natural AAV capsid variants, current strategies require virus doses of up to approximately 2 × 10^14^ vg/kg body weight (28, 36, 37). By performing intracardiac injection, we were previously able to obtain high local editing efficiency of approximately 85% at the cDNA level with an AAV9 dose of 1.5 × 10^12^ vg/kg body weight (10). Utilizing the engineered MyoAAV 2A vector enabled further reduction of the virus dose by 10-fold to 1.5 × 10^11^ vg/kg body weight, while maintaining a similar high editing efficiency. The substantially reduced viral dose and the use of an engineered AAV capsid with higher muscle tropism and lower liver tropism decrease the risk of liver toxicity or AAV-related immunological effects (28, 35, 38).

CRISPR-Cas9 gene editing to render CaMKIIδ insensitive to oxidative activation overcomes many of the challenges of traditional compound-based strategies. Careful design of a sgRNA corresponding to the *CAMK2D* gene substantially reduces the risk of targeting other enzymes or ion channels. Utilizing a cardiomyocyte-specific promoter (e.g., the troponin T promoter) to drive the expression of the gene editing components exclusively in cardiomyocytes prevents gene editing in organs other than the heart. This decreases the risk of adverse side effects, which are frequently observed with common heart failure medications. Administration of 1 *CAMK2D* editing dosage on the day of cardiac injury was sufficient to confer sustained cardioprotection over the entire observation period of 5 weeks, thereby overcoming the requirement of daily administration. As CRISPR-Cas9 gene editing is permanent, the beneficial effects are expected to be maintained longer than 5 weeks (39). This issue, as well as applying *CAMK2D* editing in a setting of a more severe IR injury, will be tested in future studies.

Identifying the optimal time point to administer the gene editing components will be critical as *CAMK2D* editing is unlikely to convey therapeutic benefits once cardiomyocytes have died. We administered the *CAMK2D* editing components immediately after reperfusion, but it will be important to test whether administration at later time points is still beneficial, since a myocardial infarction is not always diagnosed and treated immediately. In this study, we opted for an intracardiac injection of the editing components, which enabled substantial reduction of the viral dose. In the clinic, intracardiac injection of the editing components could be achieved with catheter techniques in conjunction with coronary angiography and revascularization of the infarct artery, which might incur other challenges. Thus, it will be imperative to also explore other delivery modalities like intravenous injections. Another potential limitation of CRISPR-Cas9 gene editing is its irreversibility. However, permanent silencing of a pathomechanism might be suitable for patients with chronic diseases (e.g., coronary artery heart disease), where chronic disturbance of a pathogenic signaling cascade is perpetuated for many years. Future studies will aim to test additional nonviral delivery strategies and to determine whether *CAMK2D* editing is also beneficial to a broader range of cardiovascular diseases, as oxidized CaMKIIδ has been linked to numerous disorders like atrial fibrillation, diabetes mellitus, and sleep-disordered breathing (3, 15, 16, 40).

## Methods

### Plasmids.

Plasmids were ordered from Addgene and adapted using oligonucleotides (IDT) or PCR product template sequences (PrimeStar GXL Polymerase, Takara), as appropriate. NEBuilder HiFi DNA Assembly (NEB) was used to clone oligonucleotides and PCR products into restriction enzyme–digested vectors.

SgRNAs were cloned into a pmCherry_gRNA plasmid containing a U6-driven sgRNA scaffold and a cytomegalovirus-driven (CMV-driven) pmCherry fluorescent protein (gift from Ervin Welker, Research Centre for Natural Sciences of the Hungarian Academy of Sciences, Budapest, Hungary; Addgene plasmid 80457) (10). ABE8e(TadA-8e V106W) was a gift from David Liu (Harvard University, Cambridge, Massachusetts, USA; Addgene plasmid 138495) (19). pCMV-T7-ABEmax(7.10)-SpRY-P2A-EGFP (RTW5025) was a gift from Benjamin Kleinstiver (Massachusetts General Hospital, Boston, Massachusetts, USA; Addgene plasmid 140003) (21). ABE8e(TadA-8e V106W)-SpRY was obtained by adapting pCMV-T7-ABEmax(7.10)-SpRY-P2A-EGFP (RTW5025). Other plasmids used to produce AAVs are described in the corresponding paragraph.

### Human iPSCs.

Human iPSCs were previously generated and used in our laboratory (10, 24). We used Matrigel-coated 6-well polystyrene culture plates (Corning) to maintain iPSCs in mTeSR1 media (STEMCELL). IPSCs were passaged at 70%–80% confluency using Versene (Thermo Fisher Scientific).

Approximately 8 × 10^5^ iPSCs were treated with 10 μM ROCK inhibitor (Y-27632, Selleckchem) 1 hour before the nucleofection experiments. Accutase (Innovative Cell Technologies) was used to obtain single cell status. We mixed the iPSCs either with 1.5 μg of pmCherry_gRNA plasmid carrying sgRNA1 and 4.5 μg ABE8e(TadA-8e V106W) plasmid or with 1.5 μg of pmCherry_gRNA plasmid carrying sgRNA2 and 4.5 μg ABE8e(TadA-8e V106W)-SpRY plasmid. iPSCs were nucleofected using the P3 Primary Cell 4D-Nucleofector X Kit (Lonza), according to the manufacturer’s protocol. The culture media was then supplemented for 1 day with ROCK inhibitor (10 μM) and Primocin (100 μg/mL) (InvivoGen). Using fluorescence-activated cell sorting, we collected pmCherry-positive cells 2 days after the nucleofection experiment.

### Off-target analyses in human iPSCs.

The editing efficiency was assessed in human iPSCs after nucleofection of ABE components and either sgRNA1 or sgRNA2. The cutting frequency determination (CFD) score of CRISPOR was used to identify the top 8 genomic sites for potential off-target editing in the human genome for both ABE8e(TadA-8e V106W) + sgRNA1 and ABE8e(TadA-8e V106W)-SpRY + sgRNA2 (10, 41). The predicted sites for sgRNA2 have been analyzed previously following nucleofection together with ABE8e-SpRY (10). ABE8e(TadA-8e V106W)-SpRY is a modified adenine base editor and less prone to potential off-target editing (10, 19).

Genomic DNA was isolated using DNeasy Blood & Tissue Kit (Qiagen) and we PCR-amplified the targets using PrimeStar GXL Polymerase (Takara, primers listed in Supplemental Table 1). In a second PCR round, we added the Illumina flow cell binding sequences and barcodes. Afterward, we purified the PCR products with AMPure XP Beads (Beckman Coulter), tested them for integrity on a 2200 TapeStation System (Agilent), and measured the DNA concentration using a QuBit dsDNA high-sensitivity assay (Invitrogen). After sample pooling and sequencing by an Illumina MiSeq, we demultiplexed the samples and analyzed the amplicon reads using CRISPResso2 (42).

We reported the background-corrected adenine-to-guanine editing efficiency for each adenine along the 20-base pair target DNA sequence corresponding to either sgRNA1 or sgRNA2.

### Generation of a humanized CAMK2D knockin mouse model.

To be able to use the sgRNAs optimized for the human genome, we humanized the regulatory domain of CaMKIIδ in mice, which is encoded by exons 11 and 12. Therefore, we replaced 1,386 base pairs (300 base pairs of the 3′ end of intron 10, 84 base pairs of exon 11, 459 base pairs of intron 11, 43 base pairs of exon 12, and 500 base pairs of the 5′ end of intron 12) of the mouse *Camk2d* gene with the corresponding human sequence using CRISPR-Cas9-mediated HDR. We used 5′- and 3′-homology arms of 1,200 base pairs corresponding to the mouse genome (Supplemental Figure 1). The HDR template was obtained by PCR amplification of the corresponding mouse and human genomic segments and cloning into a plasmid backbone (Supplemental Table 2). We designed 2 sgRNAs that corresponded to mouse genomic segments of either intron 10 or intron 12 and were both within the region that was later replaced with the human sequence (Supplemental Table 2). The sgRNAs were ordered and synthesized from IDT.

We injected both sgRNAs (each 15 ng/μL), the HDR template (12.5 ng/μL), and Cas9 mRNA (50 ng/μL, TriLink BioTechnologies) into the pronucleus and cytoplasm of mouse zygotes to humanize the regulatory domain of CaMKIIδ. We treated 6-week-old C57BL/6N (Charles River Laboratories) female mice for superovulation and mated them with C57BL/6N stud males to induce zygote production. After that, we isolated zygotes, transferred them to M16 (Brinster’s medium for ovum culture supplemented with 100 units/mL penicillin and 50 mg/mL streptomycin), and injected in M2 medium (M16 medium and 20 mM HEPES). After culture in M16 medium for 1 hour at 37°C, we transferred the injected zygotes into the oviducts of pseudo-pregnant female ICR mice.

For genotyping, we selected 2 primers that were both outside the homology arms of the template (Supplemental Table 1). Using these primers, we PCR amplified ear genomic DNA and digested the PCR product with SbfI (NEB). Since there is no SbfI restriction site in the WT PCR product, there was a single product of 3,898 base pairs (Supplemental Figure 1B). In contrast, the PCR product of humanized *CAMK2D* knockin mice contains 1 restriction site for SbfI, resulting in 2 products of 2,473 and 1,432 base pairs (Supplemental Figure 1B). Since the human intron 11 is 7 base pairs longer than the mouse intron 11, the undigested PCR product of humanized *CAMK2D* knockin mice is slightly longer (3,905 base pairs) than that of WT mice. One mouse of the F_0_ generation with a high knockin level was selected as a founder for the humanized *CAMK2D* knockin line and backcrossed for at least 3 generations. Successful integration of the knockin in the genome as well as successful transcription and splicing were confirmed by Sanger sequencing of the DNA and cDNA (after reverse-transcription PCR), respectively.

### Virus production.

To deliver the optimized gene editing constructs in vivo, we used AAV with the AAV2/MyoAAV 2A serotype (28). Since an ABE system exceeds the packaging limit of AAV, we designed a split-virus system encoding the N- and C-terminal halves of the base editor, as previously described (10, 24). Utilizing a split-intein trans-splicing system enabled reassembly of both parts to a functional ABE system in vivo. Therefore, we adapted the N- and C-terminal ABE constructs from Cbh_v5 AAV-ABE N-terminal (gift from David Liu, Harvard University, Cambridge, Massachusetts, USA; Addgene plasmid 137177) (43) and Cbh_v5 AAV-ABE C-terminal (gift from David Liu, Harvard University, Cambridge, Massachusetts, USA; Addgene plasmid 137178) (43), respectively. The modified plasmids carried either ABE8e(TadA-8e V106W) combined with sgRNA1 or ABE8e(TadA-8e V106W)-SpRY combined with sgRNA2. A cTnT promoter was used to drive the expression of the base editors exclusively in cardiomyocytes. The expression of the sgRNAs was driven by a U6 promoter.

To produce AAVs, near-confluent HEK293T cells (ATCC) were transfected with 6 μg of either AAV-ABE plasmid, 12 μg of pHelper plasmid (Cell Biolabs), and 24 μg of pRepCap Myo2A plasmid (provided by Jan-Bernd Funcke, University of Texas Southwestern Medical Center). Transfection was performed in DMEM supplemented with 5% FBS, 2 mM L-alanyl-L-glutamine dipeptide (GlutaMAX), 100 U/mL penicillin, and 100 mg/mL streptomycin, utilizing PEI (linear, MW25000; Polysciences) at a PEI:DNA mass ratio of 3:1. After 3 days, culture supernatant was harvested and stored at 4°C. Fresh DMEM containing 5% FBS, 2 mM GlutaMAX, 100 U/mL penicillin, and 100 mg/mL streptomycin was then replenished. Five days following the transfection, both culture supernatants and cells were collected, combined with the stored supernatants, and subjected to centrifugation to isolate the cellular pellets.

AAVs were purified according to a recognized 3-phase partitioning technique (44). The separated supernatants were reserved, while cells were lysed using a buffer composed of 50 mM Tris-HCl, 150 mM NaCl, and 2 mM MgCl_2_ at pH 8.0. Lysis was achieved through a series of 3 freeze-thaw cycles involving liquid nitrogen and a 37°C water bath. The resulting cell lysates were supplemented with 50 U/mL of Benzonase (Sigma-Aldrich) and 10 U/mL of RNase I (Thermo Fisher Scientific). Following 30 minutes of incubation at 37°C, 0.5% (w/v) SDS was introduced, and the mixture was incubated for another 30 minutes at 37°C. Debris was eliminated through centrifugation from the lysates, which was then merged with the previously separated supernatants to which 500 mM NaCl and 8% (w/w) PEG-8000 were added. After incubation at 4°C overnight and subsequent centrifugation at 4,000*g* and 4°C for 30 minutes, the ensuing pellets were resuspended in purification buffer II (50 mM Tris-HCl, 500 mM NaCl, 2 mM MgCl_2_, 1% (w/w) sarkosyl, and 1% (v/v) Triton X-100 at pH 7.5). Samples were saturated with (NH_4_)_2_SO_4_ at 20% and incubated for 5 minutes at 37°C and 300 rpm. After that, tert-butanol was added, samples were incubated for another 5 minutes at 37°C and 300 rpm, and subsequently centrifuged for 10 minutes at 4,000*g* and room temperature to facilitate collection of the lower aqueous phases. Amicon Ultra-15 centrifugal filter units with a molecular weight cut-off of 100 kDa (Thermo Fisher Scientific) were prewashed with DPBS containing 0.01% (w/v) Pluronic F-68 and used for the following step. The collected aqueous phases were subjected to 3 washing steps with an excess of injection buffer (DPBS containing 200 mM NaCl and 0.001% (w/v) Pluronic F-68) prior to concentration. The resulting purified AAVs were divided into aliquots and stored at –80°C until use. AAV titers were quantified with qPCR according to a recognized protocol (primers listed in Supplemental Table 1) (45).

### IR injury.

All mice were housed and bred at the Animal Resource Center at the UT Southwestern Medical Center, which is a pathogen-free facility with regular 12 hour light/dark cycle (temperature of 18°C –24°C and humidity of 35%–60%). There was a maximum of 5 mice per cage with ad libitum access to food and water. All mice were monitored daily for potential health problems and all mice received standard chow (2916 Teklad Global).

For all experiments, we used female and male mice that were homozygous for the humanized *CAMK2D* knockin. IR surgery was performed in 12-week-old mice, as previously described (10). Ketamine/Xylazine complex was used for anesthesia. Mice were intubated and ventilated with a MiniVent mouse ventilator (Hugo Sachs Elektronik, 250 μL stroke volume, 105 breaths/min). The body temperature was monitored with a rectal probe and kept close to 37.0°C. After opening the chest between the left fourth and fifth ribs, a 7-0 nylon suture was put below the left anterior descending coronary artery, and a nontraumatic occluder was placed on the artery. After 45 minutes of ischemia, the suture and occluder were removed (reperfusion) and we injected the MyoAAV 2A carrying the CRISPR-Cas9 components directly into the left anterior wall of the heart, which is the area of injury following ligation of the left anterior descending coronary artery.

All mice were injected with a total virus dose of 1.5 × 10^11^ vg/kg body weight that was diluted with 0.9% sodium chloride solution (Sigma-Aldrich) to an injection volume of 30 μL. Control mice received either only the N- or only the C-term of the virus, which has previously been shown to be a suitable nonediting control virus (IR+control virus) (10). Mice subjected to *CAMK2D* editing received equal amounts of N- and C-term with either sgRNA1 (IR+sgRNA1) or sgRNA2 (IR+sgRNA2). Sham-treated mice were subjected to 45 minutes open chest without IR and without any injection (sham). All surgeries and intracardiac injections were performed by the same experienced surgeon in a standardized manner and blinded to the content of liquid (control virus, sgRNA1 or sgRNA2). Mice were euthanized after 5 weeks for further histological and molecular analyses.

### Echocardiography.

Cardiac function was assessed by 2-dimensional transthoracic echocardiography (Vevo2100 imaging system, VisualSonics) in conscious mice 1 week before as well as 24 hours and 3 weeks after the surgery. M-mode traces were acquired to average 3 consecutive heart beats. LVIDd and end-systolic (LVIDs) internal diameter were analyzed to calculate fractional shortening (%) using the equation [(LVIDd – LVIDs) / LVIDd] × 100. All echocardiographic measurements were performed and analyzed by the same experienced investigator, who was blinded to the treatment group.

### Treadmill exhaustion test.

Exercise capacity was investigated 4 weeks after the IR injury (without or with editing *CAMK2D*) on an Exer-3/6 rodent treadmill with 10° inclination (Columbus Instrument) (10, 46). There was an electric shock grid at the rear end with a stimulation intensity of 10 at a frequency of 3 Hz. All mice were acclimated to the treadmill on 3 consecutive days by subjecting them to 10 minute sessions with a treadmill velocity of 0, 5, and 10 m/min for the first, second, and third day, respectively. After a warm-up of 10 m/min for 2 minutes, the velocity was set to 15 m/min. The velocity was accelerated at a rate of 0.6 m/min per minute until the mouse was exhausted, which was defined by continuous standing for 5 seconds on the electrical shock grid. All treadmill exhaustion tests were performed by the same investigator, who was blinded to the surgery (sham versus IR) and the treatment (control virus versus sgRNA1 versus sgRNA2).

### Western blot analysis.

For Western blot analysis, snap-frozen mouse cardiac tissue was pulverized using a tissue crusher and proteins were isolated with RIPA buffer (Sigma-Aldrich) supplemented with protease- and phosphatase-inhibitors (Roche). After the genomic DNA was broken by sonication with a Bioruptor Pico (10 on/off-cycles of 30 seconds sonication, Diagenode), we centrifuged the samples at 4°C for 15 minutes at 10,000*g*. The protein concentration was measured by a BCA assay (Thermo Fisher Scientific) and equal amounts were loaded on a Mini-PROTEAN TGX gel (Bio-Rad). Proteins were transferred onto a polyvinylidene fluoride membrane (Millipore), blocked in 5% milk supplemented with TBS-Tween 0.1%, and incubated with the primary antibody at 4°C overnight. Primary antibodies were rabbit polyclonal anti-oxCaMKII (1:1,000, Sigma-Aldrich, catalog number 07-1387), mouse monoclonal anti-CaMKII (1:1,000, BD Biosciences, catalog number 611293), and mouse monoclonal anti-GAPDH (1:1,000, Sigma-Aldrich, catalog number MAB374). Afterward, membranes were incubated for 1 hour at room temperature with either HRP-conjugated goat anti-rabbit (1:10,000, Bio-Rad, catalog number 1706515) or HRP-conjugated goat anti-mouse (1:10,000, Bio-Rad, catalog number 1706516), which were used as secondary antibodies. Immunodetection was performed in the presence of Western Blotting Luminol Reagent (Santa Cruz Biotechnology) on a ChemiDoc MP Imaging System (Bio-Rad). The densitometric analysis was performed using ImageJ.

### CaMKII activity assay.

Snap-frozen mouse cardiac tissue was pulverized using a tissue crusher and samples were lysed in a buffer containing 1% (v/v) Triton X-100, 20 mM Tris, and 100 mM NaCl supplemented with protease- and phosphatase-inhibitors (Roche) at a pH of 7.4. After centrifugation for 15 minutes at 10,000*g* at 4°C, equal volumes of the supernatant were loaded onto the CycLex CaM-kinase II assay kit (MBL International Corporation). The assay was performed according to the manufacturer’s recommendations. The absorbance was measured at a wavelength of 450 nm on a CLARIOstar microplate reader (BMG LABTECH). A standard curve with dilutions of the CaM-kinase II Positive Control (MBL International Corporation) was used to calculate the CaMKII activity of each sample, which was then normalized to the protein concentration of the lysate (BCA assay, Thermo Fisher Scientific).

### Routine histology.

Mouse hearts were carefully explanted and cleaned for 5 minutes in phosphate-buffered saline (PBS) supplemented with 0.2 M KCl for cardioplegia. After fixing in 10% neutral-buffered formalin (Sigma-Aldrich) at room temperature overnight, the samples were dehydrated in 70% ethanol, embedded in paraffin, and subjected to routine histology (picrosirius red staining). A BZ-X700 microscope (Keyence) was used to capture images of transverse cross-sections at 10× magnification (1,500 μm below the expected normoxic area). The collagen-positive area of the section was determined using ImageJ and was divided by the total area of the heart to obtain the percentage of fibrotic tissue.

### Statistics.

For this study, we used female and male mice that were homozygous for the humanized *CAMK2D* knockin. Mice were randomly assigned to the respective groups while keeping the gender distribution within the groups at a balanced ratio. All experiments were conducted in replicates. We dedicated 3 mice per group to histological analyses and 5 mice per group to further molecular analyses, which has previously been shown to be a sufficient sample size to reach statistical significance (10). All data are included in this study and reported as mean ± SEM.

Distribution of the data (normal versus nonnormal) were assessed using the Shapiro-Wilk normality test. If the data were not normally distributed or if the sample size was too small to assess normality, nonparametric tests were applied. When comparing 2 groups, 2-tailed Student’s *t* or Mann-Whitney test were applied for either normally or not normally distributed data, respectively. 1-way ANOVA with Holm-Šidák’s posthoc correction was applied for the comparison of more than 2 groups and a variable that was normally distributed. The Kruskal-Wallis test with Dunn’s posthoc correction was used for the comparison of more than 2 groups and a variable that was not normally distributed. Posthoc multiple comparisons were only performed when the ANOVA/Kruskal-Wallis test was significant. Linear regression analysis was used to test for correlations. Statistical comparisons were performed using GraphPad Prism 10 and 2-sided *P* values below 0.05 were considered statistically significant.

### Study approval.

All iPSC experiments complied with the regulations of the UT Southwestern Stem Cell Research Oversight Committee. Animal work described in this manuscript has been approved and conducted under the oversight of the UT Southwestern Institutional Animal Care and Use Committee.

### Data availability.

All data from this study are available either in the main manuscript or the supplemental material. Supporting data values associated with the main manuscript and supplemental material can be found in the Supporting Data Values file.

## Author contributions

SL and ENO conceptualized the overall objective of this study and designed the experiments. LGS and PES produced the MyoAAV 2A. SL, XMC, DA, WT, HL, and JRM performed the experiments. SL, WT, KC, and LX analyzed the data. SL wrote the initial version of the manuscript that was reviewed and edited by LGS, PES, NL, RBD, and ENO.

## Supplementary Material

Supplemental data

Supporting data values

## Version 1 10/19/2023

In-Press Preview

## Version 2 01/02/2024

Electronic publication

## References

[1] Virani SS (2021). Heart disease and stroke statistics-2021 update: a report from the American Heart Association. Circulation.

[2] Lebek S (2020). Enhanced CaMKII-dependent late INa induces atrial proarrhythmic activity in patients with sleep-disordered breathing. Circ Res.

[3] Luo M (2013). Diabetes increases mortality after myocardial infarction by oxidizing CaMKII. J Clin Invest.

[4] Nassal D (2020). Challenges and opportunities for therapeutic targeting of calmodulin kinase II in heart. Front Pharmacol.

[5] Pellicena P, Schulman H (2014). CaMKII inhibitors: from research tools to therapeutic agents. Front Pharmacol.

[6] Fischer TH (2014). Ca(2+) /calmodulin-dependent protein kinase II equally induces sarcoplasmic reticulum Ca(2+) leak in human ischaemic and dilated cardiomyopathy. Eur J Heart Fail.

[7] Toischer K (2010). Differential cardiac remodeling in preload versus afterload. Circulation.

[8] Lebek S (2021). Abnormal P-wave terminal force in lead V_1_ is a marker for atrial electrical dysfunction but not structural remodelling. ESC Heart Fail.

[9] Backs J (2009). The delta isoform of CaM kinase II is required for pathological cardiac hypertrophy and remodeling after pressure overload. Proc Natl Acad Sci U S A.

[10] Lebek S (2023). Ablation of CaMKIIδ oxidation by CRISPR-Cas9 base editing as a therapy for cardiac disease. Science.

[11] Ling H (2013). Ca2+/Calmodulin-dependent protein kinase II δ mediates myocardial ischemia/reperfusion injury through nuclear factor-κB. Circ Res.

[12] Lebek S (2018). The novel CaMKII inhibitor GS-680 reduces diastolic SR Ca leak and prevents CaMKII-dependent pro-arrhythmic activity. J Mol Cell Cardiol.

[13] Pabel S (2022). Effects of atrial fibrillation on the human ventricle. Circ Res.

[14] Beckendorf J (2018). Physiological and unappreciated roles of CaMKII in the heart. Basic Res Cardiol.

[15] Hegner P (2023). CaMKII-dependent contractile dysfunction and pro-arrhythmic activity in a mouse model of obstructive sleep apnea. Antioxidants (Basel).

[16] Purohit A (2013). Oxidized Ca(2+)/calmodulin-dependent protein kinase II triggers atrial fibrillation. Circulation.

[17] Erickson JR (2008). A dynamic pathway for calcium-independent activation of CaMKII by methionine oxidation. Cell.

[18] Liu N, Olson EN (2022). CRISPR modeling and correction of cardiovascular disease. Circ Res.

[19] Richter MF (2020). Phage-assisted evolution of an adenine base editor with improved Cas domain compatibility and activity. Nat Biotechnol.

[20] Gaudelli NM (2017). Programmable base editing of A•T to G•C in genomic DNA without DNA cleavage. Nature.

[21] Walton RT (2020). Unconstrained genome targeting with near-PAMless engineered CRISPR-Cas9 variants. Science.

[22] Komor AC (2016). Programmable editing of a target base in genomic DNA without double-stranded DNA cleavage. Nature.

[23] Koblan LW (2018). Improving cytidine and adenine base editors by expression optimization and ancestral reconstruction. Nat Biotechnol.

[24] Chemello F (2021). Precise correction of Duchenne muscular dystrophy exon deletion mutations by base and prime editing. Sci Adv.

[25] Chai AC (2023). Base editing correction of hypertrophic cardiomyopathy in human cardiomyocytes and humanized mice. Nat Med.

[26] Hinderer C (2018). Severe toxicity in nonhuman primates and piglets following high-dose intravenous administration of an adeno-associated virus vector expressing human SMN. Hum Gene Ther.

[27] Boutin S (2010). Prevalence of serum IgG and neutralizing factors against adeno-associated virus (AAV) types 1, 2, 5, 6, 8, and 9 in the healthy population: implications for gene therapy using AAV vectors. Hum Gene Ther.

[28] Tabebordbar M (2021). Directed evolution of a family of AAV capsid variants enabling potent muscle-directed gene delivery across species. Cell.

[29] Nishiyama T (2022). Precise genomic editing of pathogenic mutations in *RBM20* rescues dilated cardiomyopathy. Sci Transl Med.

[30] Maier LS (2009). Role of CaMKII for signaling and regulation in the heart. Front Biosci (Landmark Ed).

[31] Uhlen M (2015). Proteomics. Tissue-based map of the human proteome. Science.

[32] Reichart D (2023). Efficient in vivo genome editing prevents hypertrophic cardiomyopathy in mice. Nat Med.

[33] Cheng Q (2020). Selective organ targeting (SORT) nanoparticles for tissue-specific mRNA delivery and CRISPR-Cas gene editing. Nat Nanotechnol.

[34] Banskota S (2022). Engineered virus-like particles for efficient in vivo delivery of therapeutic proteins. Cell.

[35] Weinmann J (2020). Identification of a myotropic AAV by massively parallel in vivo evaluation of barcoded capsid variants. Nat Commun.

[36] Duan D (2018). Systemic AAV micro-dystrophin gene therapy for duchenne muscular dystrophy. Mol Ther.

[37] Zhang Y (2022). A humanized knockin mouse model of Duchenne muscular dystrophy and its correction by CRISPR-Cas9 therapeutic gene editing. Mol Ther Nucleic Acids.

[38] Moreno AM (2019). Immune-orthogonal orthologues of AAV capsids and of Cas9 circumvent the immune response to the administration of gene therapy. Nat Biomed Eng.

[39] Karri DR (2022). Long-term maintenance of dystrophin expression and resistance to injury of skeletal muscle in gene edited DMD mice. Mol Ther Nucleic Acids.

[40] Luczak ED, Anderson ME (2014). CaMKII oxidative activation and the pathogenesis of cardiac disease. J Mol Cell Cardiol.

[41] Concordet JP, Haeussler M (2018). CRISPOR: intuitive guide selection for CRISPR/Cas9 genome editing experiments and screens. Nucleic Acids Res.

[42] Clement K (2019). CRISPResso2 provides accurate and rapid genome editing sequence analysis. Nat Biotechnol.

[43] Levy JM (2020). Cytosine and adenine base editing of the brain, liver, retina, heart and skeletal muscle of mice via adeno-associated viruses. Nat Biomed Eng.

[44] Yu Z (2020). TPP Combined with DGUC as an economic and universal process for large-scale purification of AAV vectors. Mol Ther Methods Clin Dev.

[45] Aurnhammer C (2012). Universal real-time PCR for the detection and quantification of adeno-associated virus serotype 2-derived inverted terminal repeat sequences. Hum Gene Ther Methods.

[46] Wang Q (2021). CaMKII oxidation is a critical performance/disease trade-off acquired at the dawn of vertebrate evolution. Nat Commun.

