# Supplementary material for: CRISPR-Cas9 base editing of pathogenic CaMKIIδ improves cardiac function in a humanized mouse model
Source: J Clin Invest. 2024 Jan 2;134(1):e175164. doi: 10.1172/JCI175164 (PMC10760954; doi:10.1172/JCI175164)
Supplement: Supplemental data [file jci-134-175164-s064.pdf]

Supplemental Material

CRISPR-Cas9 base editing of pathogenic CaMKIIδ improves cardiac function in a humanized mouse model

Simon Lebek[1,2,3], Xurde M. Caravia[1,2], Leon G. Straub[4], Damir Alzhanov[1,2], Wei Tan[1,2], Hui Li[1,2], John R. McAnally[1,2], Kenian Chen[5], Lin Xu[5], Philipp E. Scherer[4], Ning Liu[1,2], Rhonda Bassel-Duby[1,2], Eric N. Olson[1,2]*

[1]Department of Molecular Biology, University of Texas Southwestern Medical Center; Dallas, TX USA.

[2]Hamon Center for Regenerative Science and Medicine, University of Texas Southwestern Medical Center; Dallas, TX USA.

[3]Department of Internal Medicine II, University Hospital Regensburg; Regensburg, Germany.

[4]Touchstone Diabetes Center, University of Texas Southwestern Medical Center; Dallas, TX USA.

[5]Quantitative Biomedical Research Center, Peter O'Donnell Jr. School of Public Health, University of Texas Southwestern Medical Center; Dallas, TX USA.

*Corresponding author: Eric N. Olson, Ph.D.; Mailing address: UT Southwestern Medical Center. Department of Molecular Biology. 6000 Harry Hines Blvd. Dallas, TX 75390-9148, USA; Phone: +1 214-648-1187; Email: Eric.Olson@UTSouthwestern.edu

Supplemental Figures

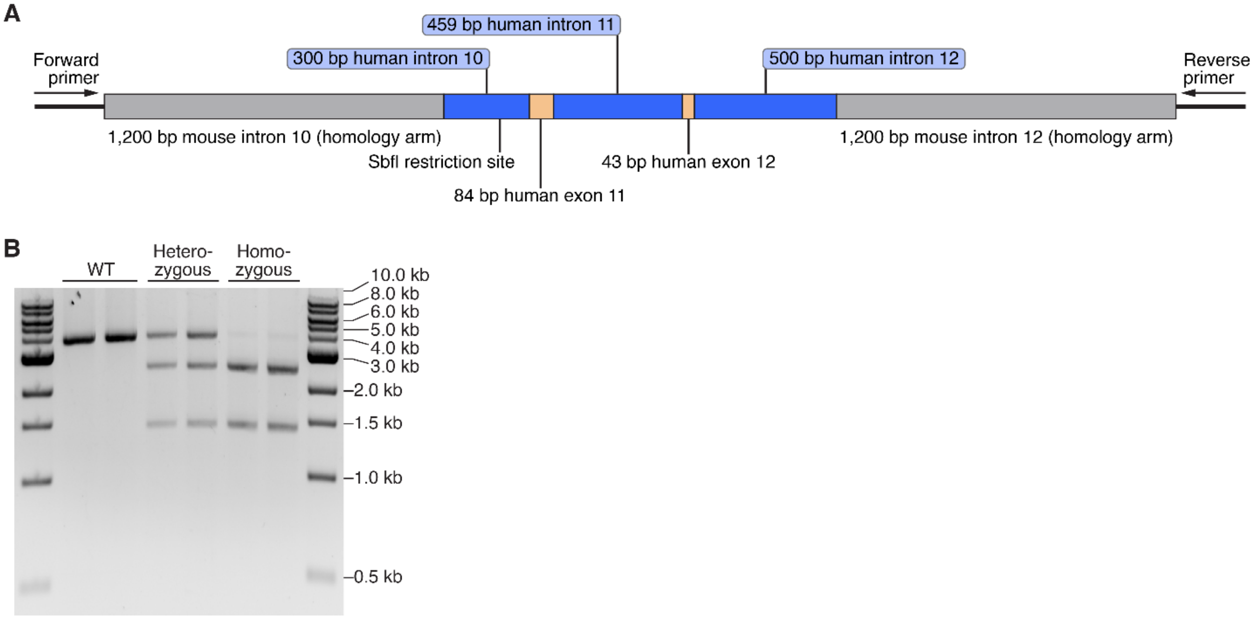

Supplemental Figure 1. Generation of a humanized *CAMK2D* knockin mouse model. A) Design of the donor template that was used to humanize the regulatory domain of CaMKIIδ. **B)** Representative genotyping gel of the SbfI-digested PCR product showing the pattern of wildtype (WT), heterozygous, and homozygous mice (n=2 each). Only the human sequence harbors a restriction site for SbfI.

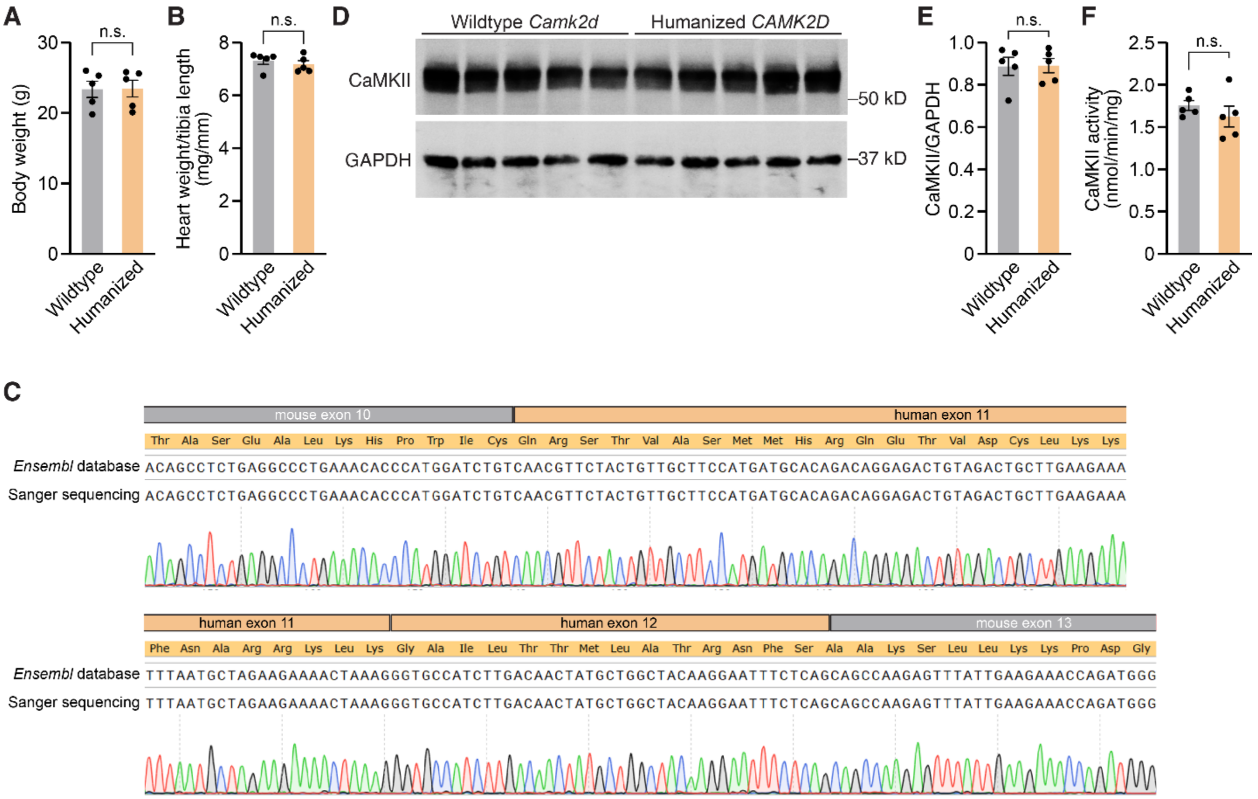

Supplemental Figure 2. Basal characterization of homozygous humanized *CAMK2D* knockin mice. A) Mean body weight for female and male wildtype and humanized mice at 12 weeks of age (n=5 per group). **B)** Mean heart weight normalized to tibia length (n=5 per group). **C)** Sequencing of cardiac cDNA of a humanized mouse and alignment with the reference sequence reveals correct splicing of the human exons 11 and 12 between mouse exons 10 and 13. **D)** Western blot analysis of total CaMKII and GAPDH in cardiac tissue of 12-weeks old mice (n=5 per group). **E)** Mean densitometric analysis of total CaMKII normalized to GAPDH (n=5 per group). **F)** Mean CaMKII activity (n=5 per group). All data are individual data points with mean ± SEM and all replicates are individual mice. Statistical comparisons are based on Student's *t* (A and E and F) or Mann-Whitney test (B); n.s. – not statistically significant.

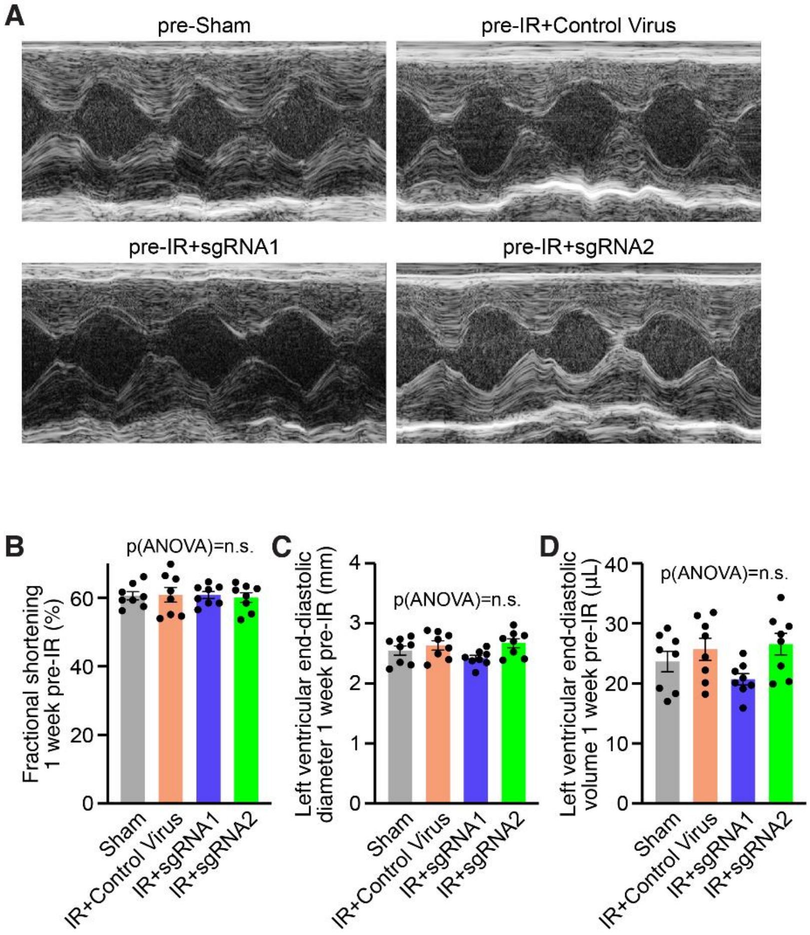

Supplemental Figure 3. Analysis of basal cardiac function and geometry one week before ischemia/reperfusion injury (IR). A) Representative M-mode traces of hearts from mice one week before either Sham, IR+Control Virus, IR+sgRNA1 or IR+sgRNA2 (echocardiography; in total n=8 per group). **B)** Mean fractional shortening one week pre-IR (n=8 per group). **C)** Mean left ventricular end-diastolic diameter one week pre-IR (n=8 per group). **D)** Mean left ventricular end-diastolic volume one week pre-IR (n=8 per group). All data are individual data points with mean ± SEM and all replicates are individual mice. Statistical comparisons are based on one-way ANOVA (B-D); n.s. – not statistically significant.

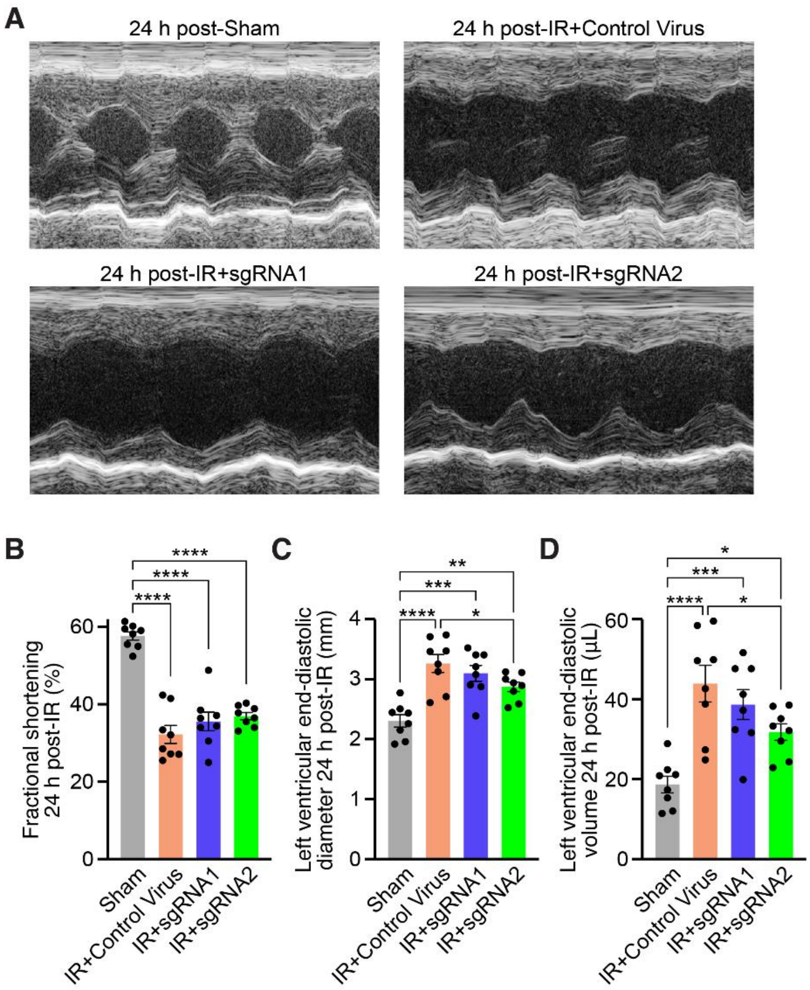

Supplemental Figure 4. Analysis of cardiac function and geometry 24 h after ischemia/reperfusion injury (IR). A) Representative M-mode traces of hearts from mice 24 h after either Sham, IR+Control Virus, IR+sgRNA1 or IR+sgRNA2 (echocardiography; in total n=8 per group). **B)** Mean fractional shortening 24 h post-IR (n=8 per group). **C)** Mean left ventricular end-diastolic diameter 24 h post-IR (n=8 per group). **D)** Mean left ventricular end-diastolic volume 24 h post-IR (n=8 per group). All data are individual data points with mean ± SEM and all replicates are individual mice. Statistical comparisons are based on one-way ANOVA post-hoc corrected by Holm-Sidak (B-D); * – $p<0.05$, ** – $p<0.01$, *** – $p<0.001$, **** – $p<0.0001$.

Supplemental Tables

Supplemental Table 1. PCR-primers used in this study.

Target	Primer	Sequence
Humanized-*CAMK2D*-mouse_ genotyping	Forward	TCTCAGTCCAGGATCCAGCCACT
	Reverse	GAATGGTGCAAAGGAACACGAGGTA
Human-*CAMK2D* (for human iPSCs and humanized mice)	Forward	TCGTCGGCAGCGTCAGATGTGTATAAGAGACAGCGTCAGTGTTGCATCTTGGT
	Reverse	GTCTCGTGGGCTCGGAGATGTGTATAAGAGACAGCCAAGAGCCCCAAAAAGAAT
Humanized-*CAMK2D*-mouse_ cDNA	Forward	TCGTCGGCAGCGTCAGATGTGTATAAGAGACAGCTGGCACACCTGGGTATCTT
	Reverse	GTCTCGTGGGCTCGGAGATGTGTATAAGAGACAGCCCATCTGGTTTCTTCAATA
sgRNA1-off-target 1	Forward	TCGTCGGCAGCGTCAGATGTGTATAAGAGACAGGGGAAGAACATCAAGCCAGA
	Reverse	GTCTCGTGGGCTCGGAGATGTGTATAAGAGACAGAATTTTGGGGGACATGAACA
sgRNA1-off-target 2	Forward	TCGTCGGCAGCGTCAGATGTGTATAAGAGACAGCCAAAAACCCAGCTTCAAAA
	Reverse	GTCTCGTGGGCTCGGAGATGTGTATAAGAGACAGTGTCAGCTTCACCCAATCAG
sgRNA1-off-target 3	Forward	TCGTCGGCAGCGTCAGATGTGTATAAGAGACAGGCAAGAAGGTTGAGCCTTTG
	Reverse	GTCTCGTGGGCTCGGAGATGTGTATAAGAGACAGGGAAAACAGCCACGATCAAT
sgRNA1-off-target 4	Forward	TCGTCGGCAGCGTCAGATGTGTATAAGAGACAGGCCATATGACCACCCATTTC
	Reverse	GTCTCGTGGGCTCGGAGATGTGTATAAGAGACAGCAACTAGGAGGAGCCACACC
sgRNA1-off-target 5	Forward	TCGTCGGCAGCGTCAGATGTGTATAAGAGACAGGCTCCTGTCTCTGCTCCTTG
	Reverse	GTCTCGTGGGCTCGGAGATGTGTATAAGAGACAGCACCTCCAAACTCACCCAGT
sgRNA1-off-target 6	Forward	TCGTCGGCAGCGTCAGATGTGTATAAGAGACAGGGCACGAGCTGTTCCAGTAT
	Reverse	GTCTCGTGGGCTCGGAGATGTGTATAAGAGACAGCCCGGTCTGCTAAGTAGTGG
sgRNA1-off-target 7	Forward	TCGTCGGCAGCGTCAGATGTGTATAAGAGACAGAAACATTCGAGATGGCCTGT
	Reverse	GTCTCGTGGGCTCGGAGATGTGTATAAGAGACAGTCCCACACACTAATGCTGGA
sgRNA1-off-target 8	Forward	TCGTCGGCAGCGTCAGATGTGTATAAGAGACAGCTGGTGGATTGCAAATTCCT
	Reverse	GTCTCGTGGGCTCGGAGATGTGTATAAGAGACAGCCCTGCTGCCCTATAGATTG
sgRNA2-off-target 1	Forward	TCGTCGGCAGCGTCAGATGTGTATAAGAGACAGATTCAGAATAGGCGGCTTCA
	Reverse	GTCTCGTGGGCTCGGAGATGTGTATAAGAGACAGGTCGTTTCCAGCCTTCATGT

sgRNA2-off-target 2	Forward	TCGTCGGCAGCGTCAGATGTGTATAAGAGACAGGTGATGGCAACACAGCAGAG
	Reverse	GTCTCGTGGGCTCGGAGATGTGTATAAGAGACAGTGGAAAAACCTCGCTCAACT
sgRNA2-off-target 3	Forward	TCGTCGGCAGCGTCAGATGTGTATAAGAGACAGGGAGGTCTGTGGGTAAAGCA
	Reverse	GTCTCGTGGGCTCGGAGATGTGTATAAGAGACAGGTATGTCCCCGGCTTACTGA
sgRNA2-off-target 4	Forward	TCGTCGGCAGCGTCAGATGTGTATAAGAGACAGACCATTGCCATTCACCTCAT
	Reverse	GTCTCGTGGGCTCGGAGATGTGTATAAGAGACAGGGCCTGCTTCCTGATTCATA
sgRNA2-off-target 5	Forward	TCGTCGGCAGCGTCAGATGTGTATAAGAGACAGACTTCAAAGTGGGGCAAGTG
	Reverse	GTCTCGTGGGCTCGGAGATGTGTATAAGAGACAGTGCAGCATTTGGAAACCATA
sgRNA2-off-target 6	Forward	TCGTCGGCAGCGTCAGATGTGTATAAGAGACAGGAAGGGGAACCAAAGGAAAG
	Reverse	GTCTCGTGGGCTCGGAGATGTGTATAAGAGACAGTGCAGGTACTCTGGCAGTTG
sgRNA2-off-target 7	Forward	TCGTCGGCAGCGTCAGATGTGTATAAGAGACAGGCCAGAGTTCATCTCCCAGA
	Reverse	GTCTCGTGGGCTCGGAGATGTGTATAAGAGACAGATGCGGGTATCATGAAATGG
sgRNA2-off-target 8	Forward	TCGTCGGCAGCGTCAGATGTGTATAAGAGACAGAATCAGTGTCAAGAGGCATCAA
	Reverse	GTCTCGTGGGCTCGGAGATGTGTATAAGAGACAGAATGACGTGGATGTGCAGAA
AAV-titer	Forward	GGAACCCCTAGTGATGGAGTT
	Reverse	CGGCCTCAGTGAGCGA

Supplemental Table 2. Sequences of sgRNAs and donor template used to humanize the regulatory domain of CaMKIIδ.

	Sequence	PAM
sgRNA-mouse-intron-10	TATACTGCTAAGTAAGGGGA	AGG
sgRNA-mouse-intron-12	ATTCATTTAGACTAATTACA	AGG
Humanization-template	ACCATGTGCTATCTACCCTTCTACACAACATGATGGGGAAATGGTTTCG<br>TGTAGGGTAGAATAAGAGATGATGACAACTCAAGATTATTTCTTTCTCTG<br>GCCAGCTCATGGGGCACCCAGTTGCAAAATCATGCCTATCTTGTCAGC<br>ATTACTTATAGTTAAGTGTTTTAGAAATTCACACATTAATATCTCAGACAT<br>GTTCACATCGAAACCAGTGGATCAAGTTAAAAATAAACATGACCAATTTT<br>ATCTTGTTAGTTATTTCTTTGTTGCAGCAGACAGTGGTATGAATTAATCT<br>ATTTTTTATATAATTTAACTTACATAATGGCAAATTTTATGAAACAATGCA<br>CTGCTTATAGAAATGAGGGATATATTTGGATTTTCTCTATTTTACTCTGT<br>GTGTGTGTGTGTGTGTGTGTGTGTGTGTGTGTGTGTGTGTGTTACTGTG<br>TGTGTGTTTTAACACAGGATCTCATGTATACCAGGTTTCCCTTGCTCTTG<br>CTATATATCTGAAGGTTGTCTAAAAGTCGTGATCCTTCCGCCTCTGCCT<br>CTCTAGTGTTGGGATTAGAGGCATGTAGGAACACGTCTAGCTTAGTGTC<br>TCTATTTTTAAAAACTCTTTACTAATTTCTATCTTTGAAGTTTCCGTCACC<br>AGAATGGGATACAGTGACACCTGAAGCCAAAGACCTCATCAACAAAATG<br>CTGACCATCAACCCTGCCAAACGTATCACAGCCTCTGAGGCCCTGAAA<br>CACCCATGGATCTGTGTAAGTCACTTTCGCATGCACCCCCAGGAGCCAA<br>ACTGAAGGATAAACCCCTAGTCACTTTTCATAAGTTTAAAATAATCTTAC<br>AGAAAAAGATGAACCTTAGTATTTAAGAAATAAATTTATAGTCGTGAAAA<br>CCACCATTGCCAGTATAGCAAACTAAACTGCCCAATTTTCTAAGTATTGT<br>CTGTAGTAAGATGAATGAGGTGTTCCTATGCCAGTAACTGATAAGACTC<br>AAGAACAGGGACTAAGATAATGACTTGAATACTTACTGCACCCCAGAAG<br>GGAAAATGTTACAAAGTCTAATTCTTTATCATTCAACAGCCTTAGAAACA<br>TGTGAGGCTTAGAAGCTAGAAGTAGTTTTAGGGGCTACACGATGGCTG<br>CAGTAGTGTGACTGTCTTGTAGAAGCCCTAAGGAAAAACCAAGGATATT<br>GTAAGAGAGTTTGctcaaactcctgacctcgtgatccacccgccttggccttccaaagtgctggaa<br>ttacaggcgtgacccaccgcgcccggccCATTTCTATACTTTCTATACGTCAGTGTTG<br>CATCTTGGTCACAAGTAGTCTTGGCAAAACTAAAATTTTATTTTTAGGTA<br>ACTTTGGCAATTGAATACTTAGAAGTTGATCCTGCAGGTGCAGCACCAT<br>TAATAAATATTTGATGAAGTCAGGGACATTATTATACTTAGTGTGTTTTAA<br>ATATTCTCAATTAAGTTACTTTTTCTCCTCTTCTTCTAGCAACGTTCTACT<br>GTTGCTTCCATGATGCACAGACAGGAGACTGTAGACTGCTTGAAGAAAT<br>TTAATGCTAGAAGAAAACTAAAGGTAAGAAATTTTACATTTTTATGGGGA<br>GGTAGATTTGACAGACTAAGAAGTGAAATACTGTGTGAGGAAAATAACT<br>TCTAGCAAAGGCTTTCAATTATGTAGACCTCAATACTCTCATTATTCTTTT<br>TGGGGCTCTTGGGAAATGGAATATACACCAGAATCAAAACTATGGTGAA<br>AAGCTGGAATCATCCTGGCAACACATGCTGTGGATCCTTCCTTCAGTGG<br>AAAACAGCTGTGCAATTTGATAATCAGAATCTGGCCCGCACACTAGGGC<br>CGTCCCAGCATCATGAGAGACAAAGGCCTTTGACTCTCAGGaaaccaaaa<br>aacaaaaacaaacaataaaaaaTCCCCTAACTGAAAGTAGGGCCAGGAAAAGA<br>TTTGTTATCAAAGACTCTATATTTGTTAGGTCCAGAATTTGCATAGGCAA<br>CACTGACTTTTGTGTTCTAATTTTTTTCACAGGGTGCCATCTTGACAACT<br>ATGCTGGCTACAAGGAATTTCTCAGGTACATGCATTGGGAACTCTGCTT<br>CTTATCCCTTGGTCTTCTTTTTAAGTAGTCTCTGTTTTTTTGTCTCTGGAA	n/a

	TTTTAAAAATCTAATAATTAGAAAAACTTCATTGTTCAGGGGTGGCAAAT<br>GTGTGGCACAGATTCCTTCTACACCTATGGCTGCCATTAGCAGTCTACC<br>TGGGTGCTGTCTGCTGCCGAGCCTCCGTGACTGAACCCCTCACAAGAC<br>TGCTTCTGGCAGCAGTGGCCACCAGTCAGTGCTGGTGCAGGACTAGCC<br>TCATCTGTCAAGTGCTGGGGTGCGCACGAGTTGTGGAAATAAATACTC<br>CCTCATTTACGATCTCAGCGTTTCAAACCTAGAATTTATTACTGATATTT<br>CATAAAGTAATAACTTTGAATAAGTTATTTTTGGTCTCATTTTCATAGGGA<br>ATCCTAGTATGCAAGATTTTTAAAAGAAGACCATTATTggccgggcgtggtggct<br>cacgcctgtaatcccagcactttgggCCACATGCGCATATGGGGATGTCATCCAATA<br>ATAGTATTCCTCAAAATGATTGCATGGGGTGGGGGGTGACAGAAGGAA<br>ACACAAAAAAAAAAAGAAACTTACATACGAGGTAAATTCAGCCAAGAAGT<br>TTCTTTTCTCTAAAAGTGACTTTCCACAATGGACGTAAAGTGTGTATTAC<br>TTCCAGGAAAGGATGCTTAAGTCTTGGAGTCCTCCCTGATAGATTATGT<br>AAAAAGCACTAGGCATTCAAAAGCCAAACGTTGGCAAGTGCTAACCTGA<br>CATATATGGAGTGGGCTGAGGCAGCAACTAAGTTTCTGCCTTATTTGAT<br>AAACTTATACTTTTGGGGAAGGCCATCAGGTGATGCATGTCCTTAGTCC<br>CAACATTCTGGAGGCAGAGGAAGGTTGAACTCTGTAAGTTCAAGGCTA<br>GCCTGCTCTACATAGAGTTTTAGGCCATTCAAGGTACATGGTGAGACCC<br>TGTTTTAAAAAGGAAGAAAGGGATGGATGAATGAAGGGAGGGAGGGAG<br>AAAGGGAGGAAGAGAGAGAGGGAAGGAGGGAAAGAGGGAGGGGAAG<br>AGGGGAGAGACAGACAGACAGATAGACTTATAGTTTAGATGTTTATTCT<br>TAGAACACTTGTCAAAAATGACATTACTTTTGTTTTGGGGTGGGGTTATG<br>CAGCTCTGACTTGGTTTGGCCCAGTCAGGATGTTAAAGTGATGGTCGG<br>GTGGCCACCATTCTAAGAGTATCAGACATTCTTCAGTCAGCTCCTAGTT<br>ACGGTTTAGACTAGTGTTTACAGAACAGTCCTCCTTCCCACTTAGAAAG<br>ATCGTGAAATGATGAGCTGGTGAGCACCAGGCACACTGTCCCCACACA<br>GTGACCAGTGCTACACATCCAGGGGCCGCACTGCCCTGCTGTTCTGTG<br>TGCTAACTGATGTTAAGTTTAGCCTAAGAAGCCATGCTGCTTGCTGCCT<br>TTGTCAAGCTTTGTAAAGACCTAGCATGAGATTGGTGTTTATGCAGGTG<br>AGCTCAATGCCAGGTCACTAAGCATGAACTGATATATACAACCTTAAAC<br>TAAATAGATATTTTACCCTGGCCCAGGACTCAACGAATCCCTAGAAATA<br>GAGGGATTTATATAGTGTATGTTGTTAATGAAGAATTTCCTTTTCAAAGA<br>TTCTCCAGAATTAAAATCATGTATTTTGTTGACATTTTAAGTGTACCT	
